# Transmission of Hepatitis C Virus Associated with Surgical Procedures — New Jersey 2010 and Wisconsin 2011

**Published:** 2015-02-27

**Authors:** Andria Apostolou, Michael L. Bartholomew, Rebecca Greeley, Sheila M. Guilfoyle, Marcia Gordon, Carol Genese, Jeffrey P. Davis, Barbara Montana, Gwen Borlaug

**Affiliations:** 1 Epidemic Intelligence Service, CDC; 2 New Jersey Department of Health; 3 Wisconsin Division of Public Health; 4 Rutgers School of Public Health, Piscataway, New Jersey; 5 Christ Hospital, Jersey City, New Jersey

Incidents of health care–associated hepatitis C virus (HCV) transmission that resulted from breaches in injection safety and infection prevention practices have been previously documented (1,2). During 2010 and 2011, separate, unrelated, occurrences of HCV infections in New Jersey and Wisconsin associated with surgical procedures were investigated to determine sources of HCV and mechanisms of HCV transmission. Molecular analyses of HCV strains and epidemiologic investigations indicated that transmission likely resulted from breaches of infection prevention practices. Health care and public health professionals should consider health care–associated transmission when evaluating acute HCV infections.

An estimated 3.2 million U.S. residents have chronic HCV infections; during 2011, approximately 16,500 acute HCV infections were diagnosed. Molecular analyses of HCV strains have enhanced investigations of health care–associated transmission (3–5) by determining the relatedness of strains infecting persons with acute and chronic HCV infection. Two investigations of HCV infection among patients who had surgical procedures highlight the potential for HCV contamination of medications or equipment, which can result in transmissions that are difficult to recognize.

## New Jersey Investigation

On March 9, 2010, a female health care worker (patient A) underwent a uterine dilation and curettage procedure at the facility where she worked. On April 14, she sought care at the same facility with jaundice, anorexia, and abdominal discomfort. Laboratory test results included a positive HCV enzyme immunoassay result confirmed by a nucleic acid amplification test for HCV RNA and a serum alanine aminotransferase level of 1,681 IU/L compared with a normal level on March 3 before surgery (normal range = 7–40 IU/L). After notification on May 6, the New Jersey Department of Health (NJDOH) investigated the potential for HCV transmission during the patient’s surgical procedure and other health care encounters; patient A reported no potential occupational exposure to HCV. The investigation included onsite inspection, staff interviews, records reviews, and observation of infection prevention practices.

**FIGURE 1:**
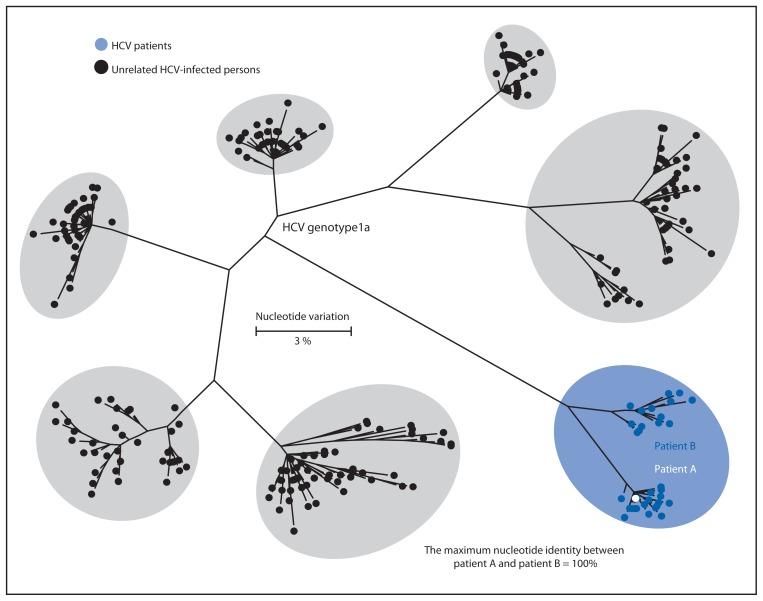
Phylogenetic tree of the E1-HVR1 genomic region of hepatitis C virus (HCV) specimens from two patients and six randomly selected unrelated controls infected with HCV genotype 1a, indicating that patient B was the likely source of patient A’s infection — New Jersey, 2010* * This maximum likelihood dendrogram was created by using the general time reversible model. Each branch represents a different viral sequence, and small distances between branches suggest genetic relatedness. The size of each oval represents the diversity of HVR1 quasispecies sequences from that specimen or group of specimens. Only unique sequence patterns are shown in the tree. For patient A, there were five total sequences; all were identical. For patient B, there were 46 total sequences, including 33 that were unique.

Review of records of all patients who had surgical procedures at the facility on March 9 before patient A’s procedure revealed one patient (patient B) with an HCV infection that had been reported to NJDOH in 2006. Blood specimens collected from patients A and B were sent to the CDC for quasispecies analysis using previously described methods (3,6,7). Results demonstrated both patients’ HCV strains were genotype 1a; 70% of chronic HCV infections are caused by genotype 1 in the United States (8). The specimens were clustered in genetic relatedness to one another with 100% identity and were distinct from control specimens collected from other persons with HCV infection (Figure 1). This indicated that patient B was the source of transmission to patient A.

Patients A and B had different surgeons, different procedures, and different operating rooms with different surgical equipment, but had the same anesthesiologist, who performed procedures that can result in HCV transmission. Following standard operating procedures at the facility, an anesthesiologist was assigned a cart and was responsible for its care and stocking. The anesthesiologist moved the cart and medications from patient to patient throughout the day. Medications were drawn into syringes and placed on the anesthesia cart surface during procedures. No policies or procedures regarding cleaning and disinfection of carts between patients existed. On March 9, the anesthesiologist treated patient B and immediately thereafter treated patient A. Propofol was the only medication common to both procedures. The anesthesiologist said there was no reuse of needles and syringes or reuse of single-dose vials; the number of vials used could not be verified by pharmacy records.

The facility provided a list of all patients treated by the anesthesiologist during 2005–2010. This list was matched to NJDOH reports of hepatitis B virus (HBV) and HCV infection. By considering the timing and sequence of patient procedures, 80 patients were identified who underwent surgical procedures after procedures on patients on NJDOH’s list of persons with known HCV infection; those 80 patients were recommended for HCV testing. No patient had a procedure after a procedure on a patient known to have HBV infection. No additional cases of HCV infection were detected from patient testing or investigation of cases reported to NJDOH’s communicable disease surveillance system

## Wisconsin Investigation

On June 1, 2011, the Wisconsin Division of Public Health (WDPH) was notified of a patient with HCV genotype 4 (HCV-4) infection. HCV-4 infections typically occur in the Middle East and Africa and are not commonly documented in Wisconsin. An investigation was conducted to identify the source and determine whether the HCV-4 infection represented a novel or persistent source of exposure.

The patient (patient 1) was an adult male with a history of type 2 diabetes, hypertension, and chronic renal disease who underwent hemodialysis for approximately 1 year until he received a single transplanted kidney on May 28, 2009, at hospital A. Routine HCV antibody testing was conducted during outpatient visits 1 year after the kidney transplantation and annually thereafter, per the transplant facility’s HCV testing protocol. Patient 1’s HCV antibody test results were reported as negative on October 13, 2008, May 28, 2009, and April 27, 2010. Occasionally, persons with chronic HCV infection, including those who are immunocompromised, are persistently anti-HCV antibody negative, and detection of HCV RNA might be the only evidence of infection (9). On May 4, 2011, the patient’s HCV enzyme immunoassay antibody test result was positive, and HCV infection was confirmed by nucleic acid amplification test.

WDPH staff members initially focused on the renal transplant procedure and contacted the United Network for Organ Sharing for donor information. The donor’s nucleic acid amplification test results for human immunodeficiency virus, HBV, and HCV were negative at the time of organ procurement. Hospital A received the single kidney for transplantation into patient 1. The United Network for Organ Sharing informed WDPH that the donor’s liver and second kidney were procured and shipped to hospital A to be transplanted into another patient (patient 2).

**FIGURE 2:**
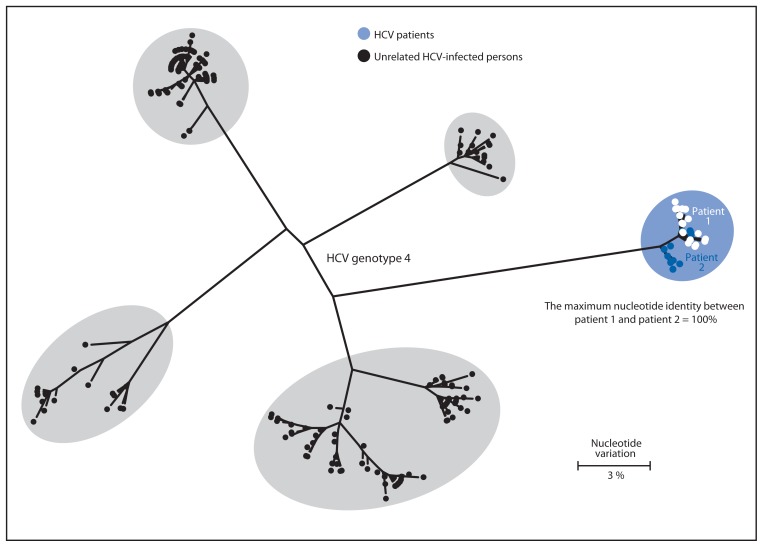
Phylogenetic tree of the E1-HVR1 genomic region of hepatitis C virus (HCV) specimens from two patients and four randomly selected unrelated controls infected with HCV genotype 4, indicating that patient 2 was the likely source of patient 1’s infection — Wisconsin, 2011* * This maximum likelihood dendrogram was created by using the general time reversible model. Each branch represents a different viral sequence, and small distances between branches suggest genetic relatedness. The size of each oval represents the diversity of HVR1 quasispecies sequences from that specimen or group of specimens. Only unique sequence patterns are shown in the tree. For patient 1, there were 25 total sequences, including 15 that were unique. For patient 2, there were 51 total sequences, including 11 that were unique.

Patient 2 was a middle-aged male with a history of liver failure resulting from chronic HCV-4 infection, chronic renal disease requiring hemodialysis, and insulin-dependent diabetes. On May 28, 2009, patients 1 and 2 had received organ transplants simultaneously in adjacent operating rooms. CDC’s quasispecies analysis of HCV-4 strains detected in blood specimens obtained from patients 1 and 2 revealed 100% identity. Laboratory and epidemiologic evidence indicated that patient 2, not the organ donor, was the likely source of patient 1’s HCV-4 infection (Figure 2).

To determine hospital care points common to patients 1 and 2 and possible modes of HCV transmission, WDPH conducted medical record reviews, onsite visits, interviews with hospital employees, and case-finding efforts. Investigation areas included the surgical intensive care unit, medical unit, inpatient dialysis unit, and related operating rooms. Organ management protocols and surgical procedures were reviewed.

The two patients’ hospital stays overlapped only during May 28–June 4, 2009, when they occupied rooms in nonadjoining areas of the surgical intensive care unit; separate health care teams were assigned to each patient. One nursing assistant likely performed vital sign assessments for both patients but did not perform invasive procedures. Multidose insulin vials were used during the two patients’ hospitalizations. However, the multidose vials remained in the medication room where doses were drawn with new needles and syringes each time and then administered in patients’ rooms. Pharmacy records indicated one possible occasion during which insulin from the same vial might have been administered to both patients. No insulin pens were used. On the day of surgery, patients 1 and 2 received hemodialysis in separate rooms in the inpatient dialysis unit and from different dialysis machines. Patient 2 was dialyzed first, 90 minutes before patient 1. Dialysis staff described how they conducted glucose testing and illustrated the correct steps for cleaning and disinfecting glucometers. No breaches in infection control practices were identified that might explain HCV transmission.

The surgical records review identified one person (surgeon 2) common to both transplant operations; all other members of the surgical teams were different. Patient 2’s transplant operation (liver and left kidney) was begun by the primary surgeon (surgeon 1). Surgeon 2 assisted on patient 2’s liver transplantation. After patient 2’s liver transplantation, surgeon 2 degowned, degloved, and left the surgical area; surgeon 2 completely changed surgical attire and rescrubbed for patient 1’s kidney transplant.

Review of the handling of donated organs indicated the liver and kidney for patient 2 were shipped separately from the kidney for patient 1. Upon arrival at hospital A, patient 2’s kidney was placed on a standard kidney perfusion machine. When patient 1’s kidney arrived later that day, both kidneys were perfused on the same machine in the operating room. Patient 2 had the first transplant operation. After patient 2’s liver was transplanted and after examining both kidneys, surgeon 1 selected the kidney to be transplanted, removed it from the perfusion machine, and placed it on the surgical field. The perfusion machine with the remaining kidney was then moved to the adjacent operating room where the kidney was transplanted into patient 1.

The Wisconsin Electronic Disease Surveillance System was searched to determine whether other HCV infections were associated with patient 1 or patient 2 or the hospital. None of the following had reported HCV infections: 162 patients hospitalized in the same units during the same period as patients 1 and 2, 10 patients who had received dialysis on the same day as the transplantation, and 124 patients who had surgical procedures at hospital A during May 28–29.

Although the precise mechanism of HCV transmission is undetermined, investigators concluded that the likely transmission venue was one surgical suite where convergence of the following events occurred and might have resulted in breaches of infection control: two kidneys were concurrently attached to the same perfusion machine; the perfusion machine was used in a blood-rich environment in patient 2’s operating room and then moved to patient 1’s operating room without cleaning or disinfection; and the kidneys were transplanted into different patients.

### Discussion

Occurrence of these two unrelated cases of health care–associated HCV infection highlights the importance of hepatitis C surveillance and investigations of possible health care transmission. During both investigations, public health authorities suspected health care transmission after reports of a single case of HCV infection, and results of quasispecies analysis provided key information for the epidemiologic investigations and helped confirm that health care exposures were responsible. Although data were limited, available evidence did not indicate an outbreak in either instance. The definitive mode of HCV transmission was not established, but both investigations highlight the probable role of contaminated equipment and supplies in bloodborne disease transmission. During both events, facility staff members transported potentially contaminated items from one procedure room to another.

After the NJDOH investigation, the New Jersey facility revised its policies and procedures regarding assigning, stocking, and cleaning anesthesia carts. Pharmacy tracking of medication vials was instituted to more accurately document the anesthesiologist’s use of each vial for each patient. All anesthesiologists were required to attend infection prevention training regarding standard precautions, injection safety, and bloodborne pathogen transmission. At the Wisconsin hospital, officials purchased a second kidney perfusion machine to eliminate the need to simultaneously perfuse multiple kidneys on the same machine.

Continuing training of all patient-care personnel and review of policies and procedures to ensure that equipment and supplies within and between procedure rooms are adequately cleaned and disinfected are important measures to optimize infection control and injection safety practices in health care settings. These cases illustrate the importance of partnerships and communication between public health and health care professionals to ensure that basic infection control and injection safety practices are optimized wherever health care is delivered.

What is already known on this topic?

Hepatitis C virus (HCV) transmission documented in health care settings has been primarily a result of unsafe injection practices including reuse of needles, fingerstick devices, and syringes, and other breaches in infection control.

What is added by this report?

Two separate occurrences of health care–associated HCV transmission likely resulted from breaches of infection prevention practices during surgical procedures. In one case, two patients received injectable propofol from the same medication cart; in the other, two patients received kidneys that had been perfused on the same machine. Molecular analyses of HCV strains helped epidemiologic investigators identify the source of transmission.

What are the implications for public health practice?

Health care and public health professionals should consider health care–associated transmission when evaluating acute HCV infections. Health care professionals should adhere to recommended standard precautions and infection control protocols to prevent transmission of bloodborne pathogens.
