# Errata

**Published:** 2015-05-22

## Vol. 64, No. 7

In the report, “Transmission of Hepatitis C Virus Associated with Surgical Procedures — New Jersey 2010 and Wisconsin 2011,” multiple errors occurred.

On page 166, in the first paragraph, the last two sentences should be replaced with the following four sentences: **“Sequences of intrahost HCV variants sampled from these two patients clustered together in the phylogenetic tree, with some variants being shared by both patients. This cluster was distinct from clusters of intrahost HCV variants obtained from control specimens collected from other unrelated persons with HCV infection. This finding indicates that both patients were infected with the same HCV strain. Phylogenetic analysis shows that sequences from patient A form a subcluster within the cluster of sequences from patient B, which suggests that patient B was the source of transmission to patient A (Figure 1).”**

On page 167, in the third paragraph, the third sentence should read, **“CDC’s quasispecies analysis showed that HCV-4 strains detected in blood specimens obtained from patients 1 and 2 shared intrahost HCV variants.”**

On page 168, the title for Figure 1 should read, **“FIGURE 1. Phylogenetic tree of the E1-HVR1 genomic region of hepatitis C virus (HCV) from two patients and six randomly selected unrelated controls infected with HCV genotype 1a, indicating that patient B was the likely source of patient A’s infection — New Jersey, 2010.”**

On page 169, the title for Figure 2 should read, “FIGURE 2. Phylogenetic tree of the E1-HVR1 genomic region of hepatitis C virus (HCV) from two patients and four randomly selected unrelated controls infected with HCV genotype 4, indicating that patient 2 was the likely source of patient 1’s infection — Wisconsin, 2011.*”

